# The complete chloroplast genome of two related fig species *Ficus squamosa* and *Ficus heterostyla*

**DOI:** 10.1080/23802359.2021.2024462

**Published:** 2022-01-24

**Authors:** Jenjira Fungjanthuek, Zheng-Ren Zhang, Yan-Qiong Peng, Jie Gao

**Affiliations:** a Chinese Academy of Sciences Key Laboratory of Tropical Forest Ecology, Xishuangbanna Tropical Botanical Garden, Chinese Academy of Sciences, Mengla, China; b College of Life Sciences, University of Chinese Academy of Science, Beijing, China

**Keywords:** *Ficus squamosa*, *Ficus heterostyla*, chloroplast genome, phylogenetic analysis

## Abstract

The *Ficus squamosa* and *Ficus heterostyla* share an undescribed pollinating fig wasp *Ceratosolen* sp. in Xishuangbanna region, which constitutes the most excellent model to study the role of convergent evolution and hybridization in the species-specific fig-wasp mutualism. The plastomes were 160,350 bp for *Ficus squamosa* and 160,300 bp for *F. heterostyla*, both in length with the typical quadripartite structure. In the two genomes, the LSC region was 88,615 bp (*F. squamosa*) and 88,535 bp (*F. heterostyla*), the SSC region was 20,071 bp (*F. squamosa*) and 20,101 bp (*F. heterostyla*), and the IR regions of both figs were 25,832 bp. They contained 113 unique genes, including a set of 78 protein-coding genes, 30 transfer RNA (tRNA) genes, four ribosomal RNA (rRNA) genes, and one pseudogene (*infA*). Phylogenetic analysis based on the complete chloroplast (cp) genomes within the *Ficus* genus suggests that they are closely related sister species.

The genus *Ficus* comprises about 750 species, distributed mainly in tropical and subtropical habitats, and remarkably diverse in Southeast Asia (Wu et al. 2004). The study of this genus is of high priority because figs are keystone resources for many organisms in tropical forests (Harrison 2005; Herre et al. 2008). Besides, as one of the most typical obligate nursery pollination mutualisms, it is fascinating to study plant–insect ecology and co-evolution (Janzen 1979; Wiebes 1979; Cruaud et al. 2012). *Ficus squamosa* Roxb. (subgenus Sycomorus; Berg and Corner 2005) is a small dioecious tree or shrub mainly distributed in the Sino-Himalayan region, including India, China, Vietnam, Laos, and Thailand, and even south to Malaysia. It is normally found abundant near the streams and river banks (Berg and Corner 2005). On the other hand, *Ficus heterostyla* Merr. (Subgenus Sycomorus; Berg and Chantarasuwan 2007) is a dioecious tree that could grow to about 10 m, generally at the secondary forest. It is distributed in Thailand, Laos, Cambodia, Vietnam, and southern China (Berg and Chantarasuwan 2007). The figs are often on an elongate leafless stolon-like branchlet which could elongate several meters away from the trunk. The two species are closely related to *Ficus hispida*, especially the *F. heterostyla* used to be considered the variant of the *F. hispida*, and was reinstated by C.C. Berg & Chantarasuwan in 2007 based on the phenotypic characteristics (Berg and Chantarasuwan 2007). The previous study reported that the sympatric distributed *F. squamosa* and *F. heterostyla* in Xishuangbanna were sharing an undescribed pollinating fig wasp *Ceratosolen sp*. (Liu et al. 2015), which could lead to potential hybridization. Therefore, the two species are interested in the study of evolution and introgression in obligate pollination systems. We want to confirm these two fig species' relationship and their relationship with *F. hispida* using the whole chloroplast (cp) genome.

For chloroplast genomic analysis, fresh and clean leaves of *F. squamosa* and *F. heterostyla* were collected from a wild individual in the Nabanhe Nature Reserve, Yunnan Province, China (22°7′48″ N, 100°39′59″ E) and the Xishuangbanna Tropical Botanical Garden (XTBG), Chinese Academy of Sciences, Yunnan Province, China (21°55′54″ N, 101°15′46″ E), respectively (sample permission was granted from the National Nature Reserve of Xishuangbanna Na-ban River; and Xishuangbanna Tropical Botanic Garden). The specimen was deposited in the Lab of Coevolution Research Group in Xishuangbanna Tropical Botanic Garden (contact person: Zheng-Ren Zhang, email: zhangzhengren@xtbg.ac.cn, under the voucher number: SQU10F, ZZRhet6). The total genomic DNA was extracted using the DNAsecure Plant kit, DP320 (TIANGEN, Beijing, China). The purified DNA of *F. squamosa* and *F. heterostyla* was fragmented to 350 bp in size for library construction, and paired-end (PE) reads of 150 bp were sequenced on the MGI DNBSEQ T7 (Annoroad, Beijing, China) and Illumina Novaseq 6000 (Berry Genomics, Beijing, China), respectively. Approximately, 45,629,184 Clean Reads in *F. squamosa* and 22,549,373 Clean Reads in *F. heterostyla* were used to assemble the complete chloroplast genome using the GetOrganelle (Jin et al. 2020). The complete genome sequences were annotated using CPGAVAS2 (Shi et al. 2019) and manually adjusted in Geneious (Kearse et al. 2012). The annotated genome sequences were deposited in the National Center for Biotechnology Information GenBank under the accession numbers OK077764 and OK077762.

The two plastomes of *Ficus* were 160,350 bp (*F. squamosa*) and 160,300 bp (*F. heterostyla*) in length with the typical quadripartite structure. The LSC region was 88,615 bp (*F. squamosa*) and 88,535 bp (*F. heterostyla*), the SSC region was 20,071 bp (*F. squamosa*), and 20,101 bp (*F. heterostyla*), and the IR regions of both *figs* were 25,832 bp. The overall GC content of two complete genomes was 35.9%, while the corresponding values of the IR, LSC, and SSC regions were 42.7%, 33.5%, and 29.0%, respectively. Furthermore, the genomes contained 113 unique genes, including a set of 78 protein-coding genes, 30 transfer RNA (tRNA) genes, four ribosomal RNA (rRNA) genes, and one pseudogene (*infA*).

To determine the phylogenetic relationship of *F. squamosa* and *F. heterostyla*, chloroplast genomes of 13 *Ficus* were selected for phylogenetic analysis, using *Morus alba* and *Antiaris toxicaria* as outgroups. The whole plastome sequences with one IR region were aligned using MAFFT (Katoh and Standley 2013), all indels were removed by trimAI (Capella-Gutiérrez et al. 2009). The maximum-likelihood (ML) tree was reconstructed by IQ-TREE (Nguyen et al. 2015) based on the TVM + G4 + F nucleotide substitution model, which was selected by modelFinder (Kalyaanamoorthy et al. 2017). The support for the phylogenetic tree was inferred by bootstrapping with 10,000 ultrafast bootstraps (Minh et al. 2013). Our result indicated that *F. squamosa* and *F. heterostyla* were closely related sister species with 100% bootstrap support (Figure 1). The complete chloroplast genome of *F. squamosa* and *F. heterostyla* will provide new information into evolutionary biology in the genus of *Ficus*.

**Figure 1.**
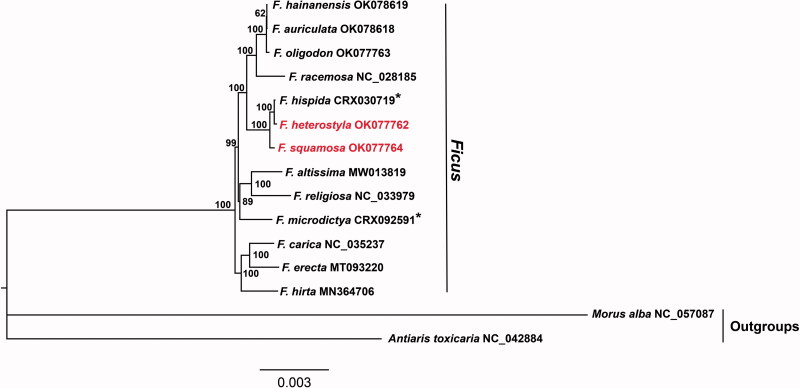
Maximum-likelihood phylogenomic tree inferred from *F. squamosa*, *F. heterostyla*, and other 11 *Ficus* species and two outgroup species using complete chloroplast genomes. Numbers at nodes correspond to ML bootstrap values based on 10,000 ultrafast bootstraps. Asterisks indicate figs chloroplast genomes were from BIG Data Center (https://bigd.big.ac.cn/gsa/) under BioProject accession number GSA: PRJCA002187.

## Authors contributions

JG and YQP conceived the study; JF and ZRZ collected the samples; JF and ZRZ analyzed the data; JG and ZRZ wrote the manuscript. All authors confirmed the final version of the manuscript.

## Data Availability

The data that support the analyses and results of this study are openly available in GenBank of NCBI at https://www.ncbi.nlm.nih.gov/ under the accession nos. OK077764 and OK077762, respectively. The associated BioProject, SRA, and Bio-Sample numbers are PRJNA763030, SRR15882953, and SAMN21421312; PRJNA763040, SRR15883057, and SAMN21421344, respectively.
